# Chronic Alcoholic Liver Disease and Mortality Risk in Spontaneous Bacterial Peritonitis: Analysis of 6,530 Hospitalizations

**DOI:** 10.7759/cureus.8189

**Published:** 2020-05-18

**Authors:** Renu Bhandari, Khalida Khaliq, Virendrasinh Ravat, Pawandeep Kaur, Rikinkumar S Patel

**Affiliations:** 1 Medicine, Manipal College of Medical Sciences, Kaski, NPL; 2 Psychiatry/Medicine, North Tampa Behavioral Health, Tampa, USA; 3 Internal Medicine, Krishna Institute of Medical Sciences, Karad, IND; 4 Medicine, Sri Guru Ram Das Institute of Medical Sciences and Research, Amritsar, IND; 5 Psychiatry, Griffin Memorial Hospital, Norman, USA

**Keywords:** spontaneous bacterial peritonitis, alcohol-related liver disease, alcohol dependence, alcohol misuse, mortality risk

## Abstract

Objective

Our study aimed to assess the risk of in-hospital mortality due to chronic alcoholic liver disease (CALD) and other comorbidities in spontaneous bacterial peritonitis (SBP) inpatients.

Methods

We conducted a cross-sectional study using the Nationwide Inpatient Sample (NIS, 2012 to 2014) from the United States and included 6,530 patients (age 18-50 years) with a primary diagnosis of SBP. Logistic regression was used to evaluate the odds ratio (OR) for in-hospital mortality in SBP by comorbidities.

Results

The prevalence of CALD in SBP patients is 43.6%, and a higher proportion were males (68.8%) and whites (67%). Middle-aged adults (OR 2.8, 95% CI 1.74-4.45) had higher odds of in-hospital mortality in SBP patients. Race and sex were non-significant predictors for mortality risk. Patients with comorbid coagulopathy (OR 1.9, 95% CI 1.45-2.48) and heart failure (OR 3.9, 95% CI 2.46-6.36) have increased mortality in SBP inpatients. After controlling confounders, CALD was significantly associated with increased in-hospital mortality (OR 1.5, 95% CI 1.12-1.94) in SBP inpatients.

Conclusion

CALD is an independent factor in increasing the risk of in-hospital mortality in SBP patients by 48%. Alcohol use screening, and alcohol abstinence and supportive therapy need to be implemented at an earlier stage to improve health-related quality of life and reduce in-hospital mortality in SBP patients.

## Introduction

Spontaneous bacterial peritonitis (SBP) is an infection of the ascitic fluid without any previous evidence of intra-abdominal surgery [1]. SBP is diagnosed based on polymorphonuclear neutrophils higher than 250 cells per cubic millimeter [2]. The prevalence of SBP in liver cirrhosis range from 11% to 14% and the incidence of SBP in hospitalized patients ranges from 10% to 30% [3,4]. In earlier days, SBP was associated with a high mortality rate of about 70%, but due to the advancement of treatment and diagnostic paracentesis, the mortality rate in hospitalized patients has decreased to around 17.6% [4]. Some of the studies have shown a higher mortality rate in older age, female sex, and patients with complications of liver cirrhosis like hepatic encephalopathy, acute kidney injury, variceal bleed, and sepsis [4].

Alcohol consumption has increased worldwide and alcohol-related complications range from hepatic steatosis to alcoholic hepatitis to alcoholic cirrhosis and end-stage liver disease [5]. It takes about 10 years to progress, so abstinence is the best way to prevent alcohol-related diseases [6]. According to the World Health Organization (WHO), alcoholism results in 3.3 million deaths every year, which is 6% of total global annual death [7]. About 10% to 20% of patients who are heavy drinkers develop alcohol-related liver diseases [8]. About 36% of patients with chronic hepatitis have alcohol dependence or abuse problems [9]. Hospital admission in alcoholic liver disease has increased by 18.3% [10] from 2012 to 2016. Chronic alcoholic liver disease (CALD) is diagnosed with a history of heavy alcohol intake; on physical examination, a patient may have signs Dupuytren's contracture, rhinophyma, spider angioma, palmar erythema, jaundice [8]. In CALD, clinical manifestation starts late and the disease spectrum range from alcoholic hepatitis to alcoholic cirrhosis [11].

Various theories are proposed for the pathogenesis of SBP. In patients with liver disease, there is an overgrowth of bacteria in the interstitial lumen [12]. Due to various structural abnormalities like vascular congestion and edema, bacterial translocation occurs in the mesenteric lymph nodes [12]. Patients with liver disease also have decreased immune function leading to bacteria overgrowth and seepage of bacteria into the ascitic fluid [12]. Due to decreased protein levels in cirrhotic patients, low complement level, and poor phagocytic activity, it leads to further growth of bacteria in the ascitic fluid [13,14]. SBP contributes about 9.8% of bacterial infection in alcoholic liver disease [14].

SBP is associated with poor prognosis [12]. Patient survival rates are 67%, 50%, and 42% in one month, six months, and one year, respectively, after an initial diagnosis of SBP [3]. SBP is one of the most common causes of bacterial death that contributes to about 33% of in-hospital mortality due to infection [3]. SBP occurs most commonly in liver cirrhosis patients [3]. CALD, alcoholic hepatitis, hepatitis B, hepatitis C, hemochromatosis, and autoimmune hepatitis cause liver cirrhosis. One of the studies showed 48% of patients with liver cirrhosis die because of CALD [8]. So there must be some association between the in-hospital mortality in SBP and CALD. The current literature is scarce and contradictory as some studies state that acute kidney injury is the most common cause of in-hospital mortality in SBP patients while some support CALD [4,15].

In our study, we utilized the inpatient data from the United States (US) hospitals: (1) to assess the differences in demographics and comorbidities seen in inpatients for SBP by the presence of comorbid CALD, and (2) to assess the risk of in-hospital mortality due to CALD and other comorbidities in SBP inpatients.

## Materials and methods

Data source

We utilized the Nationwide Inpatient Sample (NIS) data from 2012 to 2014 in our cross-sectional data analysis study. The NIS provides patient health information from about 4,400 non-federal hospitals across 44 states in the US [16]. Diagnostic information in the NIS is detected using the International Classification of Diseases, ninth edition (ICD-9) codes, and Clinical Classification Software (CCS) codes [17].

Inclusion criteria and outcome variables

We included adult patients (age 18 to 50 years) with a primary discharge diagnosis of SBP using ICD-9 code 567.23. These sample of 6,530 inpatients was further grouped by comorbid discharge diagnosis of CALD (N = 2,850) using the ICD-9 codes 571.2 or 571.3.

This study included the following demographic variables: age (18-35 and 36-50 years), sex (male or female), and race (white, black, hispanic, and others) [17]. The comorbid diagnosis of diabetes, hypertension, congestive cardiac failure (CCF), AIDS, renal failure, and coagulopathy were identified using ICD-9 or CCS diagnosis codes [17]. We measured the in-hospital mortality between CALD and non-CALD cohorts. In the NIS, in-hospital mortality is reported as all-cause and is the number of inpatient deaths [17].

Statistical analysis

We used cross-tabulation and descriptive statistics to discern the differences in demographics and comorbidities in SBP inpatients by comorbid CALD. We used a separate model of descriptive statistics to evaluate the in-hospital mortality by comorbidities. Logistic regression analysis was used to measure the odds ratio (OR) association of CALD with in-hospital mortality in SBP inpatients, and the model was controlled for demographic and other chronic comorbidities. A P-value of less than 0.01 was used to determine the statistical significance in all analyses and was conducted using the Statistical Package for the Social Sciences (SPSS), version 26 (IBM Corporation, Armonk, NY).

Ethical approval

To protect the patient's identity and their health information, the NIS used separate codes to de-identify the database. We do not require approval from the institutional review board [16].

## Results

We analyzed a total sample of 6,530 inpatients hospitalized for SBP, with 43.6% having comorbid CALD. The majority of the inpatients with CALD were middle-age adults 36 to 50 years (87%) and were older than the non-CALD cohort (43.3 years vs. 40.6 years, P < 0.001). A higher proportion of CALD inpatients were male (68.8% vs. 52.6%) and white (67% vs. 51.8%) compared to the non-CALD cohort. Comorbidities were seen in a lower proportion of CALD cohort other than a statistically significant difference with comorbid coagulopathy, i.e., 51.2% in CALD versus 29.8% in the non-CALD cohort as shown in Table 1.

**Table 1:** Distribution of SBP inpatients by comorbid CALD SBP, spontaneous bacterial peritonitis; CALD, chronic alcoholic liver disease

Variable	CALD (-)	CALD (+)	Total	P-value
Total (N)	3680	2850	6530	-
Mean age, years (SD)	40.6 (8.68)	43.3 (5.82)	-	<0.001
Age at admission, in %				
18–35 years	24.9	13.0	19.7	<0.001
36–50 years	75.1	87.0	80.3	
Sex, in %				
Male	52.6	68.8	59.6	<0.001
Female	47.4	31.2	40.4	
Race, in %				
White	51.8	67.0	58.3	<0.001
Black	20.6	4.4	13.6	
Hispanic	19.0	20.3	19.6	
Other	8.6	8.3	8.5	
Comorbidities, in %				
AIDS	2.6	0.7	1.8	<0.001
Diabetes	14.5	13.2	13.9	0.110
Hypertension	40.2	27.4	34.6	<0.001
Congestive cardiac failure	8.0	3.0	5.8	<0.001
Coagulopathy	29.8	51.2	39.1	<0.001
Renal failure	29.9	11.6	21.9	<0.001
In-hospital mortality, in %	2.7	6.1	4.2	<0.001

Risk factors for in-hospital mortality in SBP

Middle-age adults (36-50 years) have 2.8 times higher odds (95% CI 1.74-4.45) compared to young adults for inpatient death during SBP management. Statistically, no significant impact was seen in in-hospital mortality based on sex distribution (P = 0.486). When compared to the whites, no other races had a statistically significant association with in-hospital mortality.

Among chronic comorbidities, CCF and coagulopathy increased the risk of in-hospital mortality in SBP inpatients by four times (95% CI 2.46-6.36) and by two times (95% CI 1.45-2.48), respectively. Hypertension and diabetes had a negative association, whereas AIDS and renal failure had a statistically non-significant association with in-hospital mortality in SBP inpatients. CALD significantly increases the risk of in-hospital mortality by 1.5 times (95% CI 1.12-1.94) compared to the non-CALD cohort after controlling for demographic and other comorbidities as shown in Table 2.

**Table 2:** In-hospital mortality risk factors in SBP inpatients SBP, spontaneous bacterial peritonitis

Variable	Logistic regression model			
	Odds ratio	95% confidence interval		P-value
		Lower	Upper	
Age at admission				
18–35 years	Reference			
36–50 years	2.78	1.74	4.45	<0.001
Sex				
Male	1.10	0.84	1.46	0.486
Female	Reference			
Race				
White	Reference			
Black	0.52	0.29	0.92	0.026
Hispanic	1.03	0.57	1.13	0.205
Other	1.03	0.66	1.60	0.895
Chronic alcoholic liver disease				
No	Reference			
Yes	1.48	1.12	1.94	0.006
Comorbidities				
No comorbidity	Reference			
AIDS	1.77	0.69	4.55	0.236
Diabetes	0.12	0.05	0.29	<0.001
Hypertension	0.48	0.33	0.69	<0.001
Congestive cardiac failure	3.96	2.46	6.36	<0.001
Coagulopathy	1.89	1.45	2.48	<0.001
Renal failure	0.90	0.59	1.37	0.623

**Figure 1:**
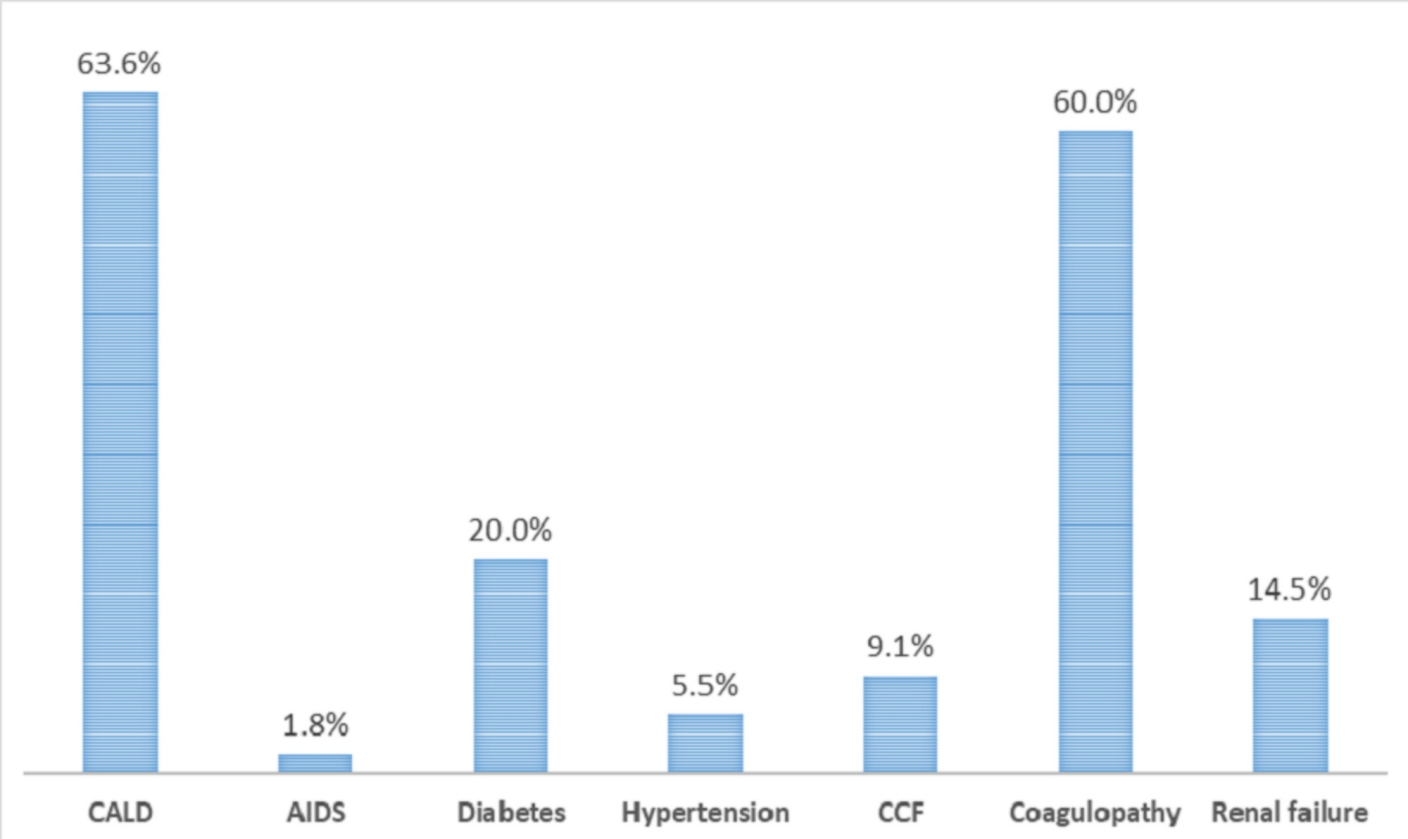
In-hospital mortality in SBP inpatients by comorbidities SBP, spontaneous bacterial peritonitis; CALD, chronic alcoholic liver disease; CCF, congestive cardiac failure

The highest numbers of death were seen in SBP inpatients with comorbid CALD (63.6%) followed by coagulopathy (60%) and diabetes (20%) as shown in Figure 1.

## Discussion

From our analysis conducted from the inpatient data from the US hospitals, the prevalence of CALD in SBP is 43.6%, and the majority of the inpatients were adults age 36 to 50 years, which was correlated to epidemiologic data of alcohol liver disease in Korea [18]. We found that middle-age adults are at a 178% higher risk of in-hospital mortality during SBP management compared to young adults. Women are at higher risk of developing CALD than men due to environmental and genetic factors [19]. Alcohol consumption and metabolism vary by gender, and so 12-22 grams/day in women and 24-46 grams/day in men may lead to the development of CALD [20]. About three-fifths of the SBP inpatients with CALD were males, which may be possible as men consume more alcohol than women [21]. We found a non-significant relationship between sex and mortality risk in SBP patients. Our study showed that about 67% of SBP patients with CALD were whites, which are possibly due to a higher consumption of alcohol in whites compared to other races [22]. There was a statistically non-significant association with mortality risk and race in our study inpatients.

Ethanol metabolism produces reactive oxygen species that in turn activate genes for lipid biosynthesis [23]. The imbalance in the central nervous system (CNS) affects the baroreceptor leading to sympathetic overactivity and stimulation of endothelin [23]. This causes renin-angiotensin activation (RAA), which leads to a decrease in left ventricular contractility [24]. In addition to this, chronic alcoholism leads to cardiomyopathy, myocyte dysfunction, affects calcium homeostasis, malnutrition hence ultimately lead to CCF [24]. Overall, CCF was seen in 5.8% of SBP inpatients and less in those with CALD (3%), but CCF was the most prominent risk factor for mortality during SBP hospitalization.

About 60% of SBP patients with CALD had comorbid coagulopathy, and it was significantly associated with in-hospital mortality and increased the risk by 89%. SBP is common in patients with cirrhosis and ascites, and cirrhotic patients are likely to have an infection that activates cytokines like tumor necrosis factor (TNF)-α, interleukin (IL)-6, and IL-1 affecting circulation and coagulation pathway [25]. Therefore, coagulopathy and renal failure further deteriorate CALD. Decompensated CALD is associated with renal failure, due to the reduction of renal blood flow, which leads to vasoconstriction of the main renal and smaller arteries, which further affects cortical ischemia [26]. One of the studies using NIS data showed a 70.3% in-hospital mortality in SBP patients due to renal failure [4]. But, after controlling for potential confounder in our study, we found a non-significant association between renal failure and mortality risk. We found no significant difference in the prevalence of diabetes between CALD and non-CALD cohorts, and both hypertension and diabetes did not have a positive association with increased in-hospital mortality risk.

Using the multivariable logistic regression model in our study, we found that CALD is significantly associated with increased in-hospital mortality in SBP by 48%. SBP most commonly occurs in a patient with cirrhosis and ascites [27]. Patients with alcoholic cirrhosis are more likely to have ascites, which could contribute to hepatic decompensation in CALD patients, thereby leading to an increase in in-hospital mortality [28].

There are some limitations to our study. Firstly, we used administrative data and so the study inpatients were included based on ICD-9 codes, which may lead to underreporting of comorbid conditions, including CALD which may affect the statistical analyses. Second, it includes all-cause in-hospital mortality and so doesn’t prove a causal relationship between mortality and comorbidities in SBP. Despite this, we have tried to strengthen our study by using national data, which represents the wide range of hospitals from 44 states across the US and the results are generalized to the inpatient population.

## Conclusions

Medical comorbidities like CCF and coagulopathy increases in-hospital mortality in SBP patients. Chronic alcohol intake leading to CALD was prevalent in SBP patients and was an independent factor that increases the risk of in-hospital mortality by 48%. Alcohol use screening, and alcohol abstinence and supportive therapy need to be implemented at an earlier stage to improve health-related quality of life and reduce in-hospital mortality in SBP patients.
